# Taxonomy of the *Nacerdes* (*Xanthochroa*) *carniolica* species-group from China (Coleoptera, Oedemeridae, Nacerdini)

**DOI:** 10.3897/zookeys.426.7449

**Published:** 2014-07-17

**Authors:** Ying Tian, Guo-Dong Ren, Qiang Li

**Affiliations:** 1 College of Plant Protection, Yunnan Agricultural University, Kunming 650201, China; 2 College of Life Sciences, Hebei University, Baoding 071002, China

**Keywords:** Coleoptera, Oedemeridae, *Nacerdes*, taxonomy, new species, China

## Abstract

This paper deals with a species-group *carniolica* of the genus *Nacerdes* from China. This species-group has seven known species/subspecies in the world and two of them are known from China. *Nacerdes* (*Xanthochroa*) *arcuata* **sp. n.** is a new species belonging to *carniolica* group. The species were collected from Anhui (Eastern China, 30°02'17.37"N, 118°50'1.72"E). A key to the species of the species-group from China is given along with a distribution map.

## Introduction

**Figure 1.**
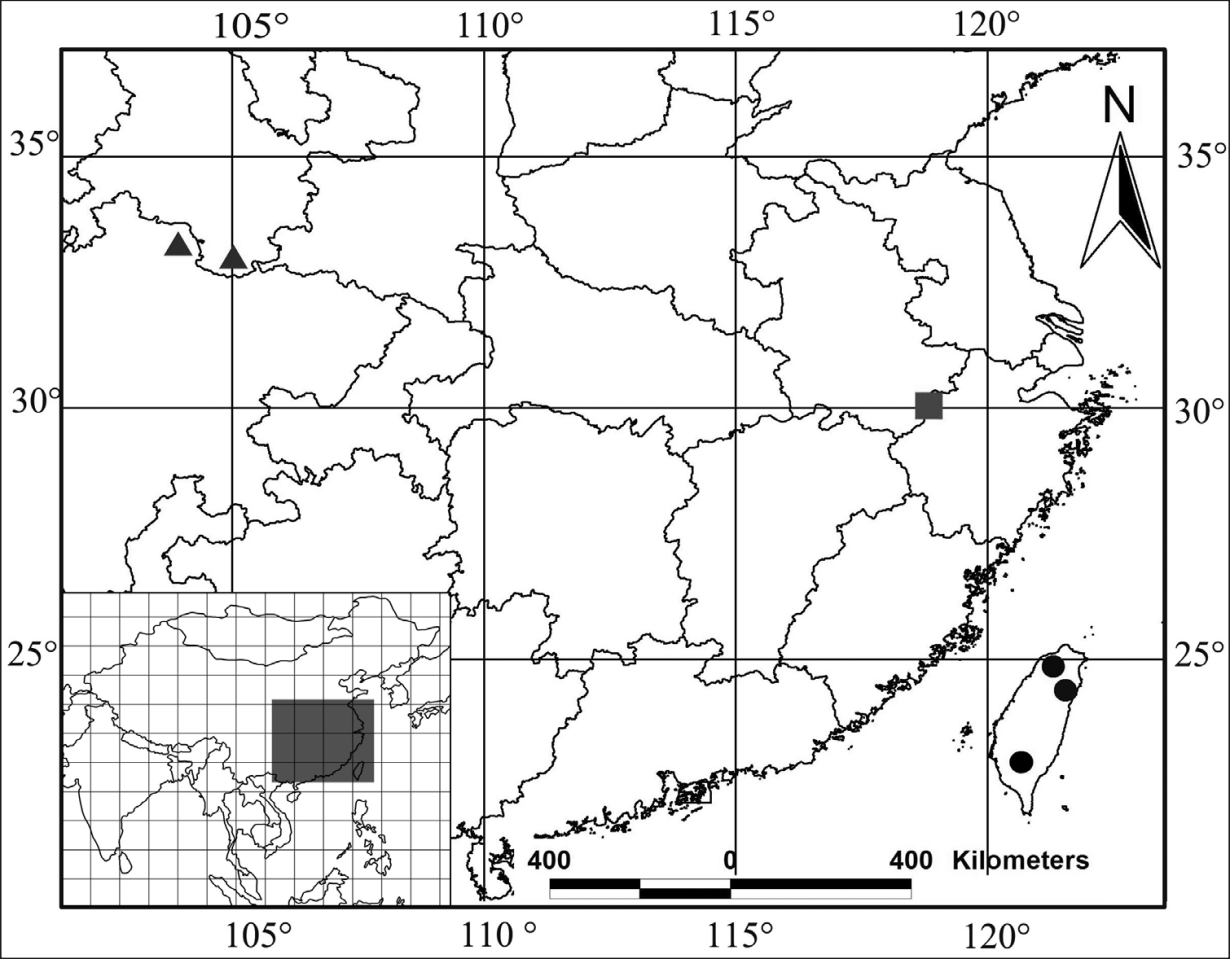
Distribution of the *Nacerdes (Xanthochroa) carniolica* species-group from China. ● *Nacerdes (Xanthochroa) hiromichii* Švihla, 2004 ▲ *Nacerdes (Xanthochroa) potanini* Ganglbauer, 1890 ■ *Nacerdes (Xanthochroa) arcuata* sp. n.

The genus *Nacerdes* was erected by Dejean (1834) for *Necydalis notata* Fabricius, 1792 (= *Cantharis melanura* Linnaeus, 1758) as the type species. More recent classifications were made by Arnett (1951, 1961), Švihla (1983, 1986, 1987, 1991, 1993, 1996, 1997, 1998, 1999, 2001, 2004, 2005, 2006, 2008, 2009), Vázquez (1993, 2002, 2006), Allemand (1993) and Yoo et al. (2008). However, Švihla (1987, 1998, 2001, 2004, 2008) made the most significant progress in *Nacerdes* identification and taxonomy, he described and recorded 22 *Nacerdes* species from China. Švihla (1998) divided the species of Western and Southern China and adjacent regions into six species-groups according to the shape of the aedeagus. Until now, the *Nacerdes (Xanthochroa) carniolica* species-group had seven species/subspecies (Švihla 1998, 2008), with two species known from China. In the present study, a new species of this group is described, an identification key to all known species of the *Nacerdes (Xanthochroa) carniolica* species-group from China is provided, and the distribution of the species-group is mapped (Fig. 1).

## Materials and methods

### Collected material

The beetles were collected by light trap from Anhui province, Eastern China region. Located across the basins of the Yangtze River and the Huai River, it borders Jiangsu to the east, Zhejiang to the southeast, Jiangxi to the south, Hubei to the southwest, Henan to the northwest, and Shandong for a tiny section in the north.

### Material identification

The collected specimens were identified based on a reference collection and key for species published by Švihla (1998).

### Examination and description

The specimens were examined and described using a Nikon (SMZ800) dissecting microscope. Examinations of aedeagus were carried out. The aedeagus was dissected under a stereoscopic microscope, cleared in 5% NaOH solution for eight minutes under water bath, then placed in a droplet of glycerol and examined under a compound light microscope. The measurements and photographs were carried out under a Leica (M205 A) dissecting microscope. A distribution map was prepared using the geographic information system software ARCVIEW GIS 3.2, based on the authors’ database of the specimens examined for this study and those mentioned in the literature. Body length is measured from the anterior margin of the clypeus to the elytral apex, body width is measured across the humeral part of elytra. All measurements are in millimeters.

The terminology used in this paper largely follows Švihla (1998).

The holotype and paratypes are deposited in MHBU – Museum of Hebei University, Baoding, China.

## Taxonomy

### *Nacerdes (Xanthochroa) carniolica* species-group

**Diagnosis.** Apicale of aedeagus with recurrent angles, so that it is arrow-shaped in dorsal view.

**Distribution.** China (Anhui, Sichuan, Gansu, Taiwan), Northern Vietnam, Europe.

### Key to the species of *Nacerdes (Xanthochroa) carniolica* species-group from China

(Adapted from Švihla 1998)

**Table:** 

1	Apex of elytra black	2
–	Elytra brown with slight metallic green tinge	*Nacerdes (Xanthochroa) potanini*
2	Aedeagal apicale short, lateral view as in Fig. 12, abruptly narrowed apically in dorsal view (Fig. 13)	*Nacerdes (Xanthochroa) hiromichii*
–	Aedeagal apicale elongate, lateral view as in Fig. 10, gradually narrowed apically in dorsal view (Fig. 11)	*Nacerdes (Xanthochroa) arcuata* sp. n.

#### Nacerdes (Xanthochroa) arcuata sp. n.

Taxon classificationAnimaliaColeopteraOedemeridae

http://zoobank.org/0DB5A33D-0E46-4DE4-A339-4A529FDBF566

Figs 2–3 , 5 –11

##### Type material.

Holotype: male (MHBU): China, Anhui Province, She County, Qingliangfeng N. R., alt. 320 m, 30°02'17.37"N, 118°50'1.72"E, 5–9.vi. 2013, Ji-Shan Xu & Cai-Xia Yuan leg. Paratypes: 2 ♂♂, 8 ♀♀ (MHBU), same data as holotype.

##### Diagnosis.

This new species belongs to the *Nacerdes (Xanthochroa) carniolica* species-group as defined by Švihla (1998) according to the shape of the aedeagus, it is similar to *Nacerdes (Xanthochroa) hiromichii* Švihla, 2004, but can be distinguished from the latter by its smaller body, black head, basal 2/3 of each femur saffron yellow, the rest of each leg sepia to black, and the different shape of aedeagal apicale.

##### Etymology.

The specific name is derived from the Latin adjective *arcuatus*, meaning arched, referring to the characteristic shape of aedeagus in this species.

##### Description.

Body length 10.3−12.7 mm, body width 2.1−2.7 mm. Head black, maxillary palpi sepia to black. Antennae black, antennomeres gradually lightening to sepia. Basal 2/3 of femora saffron yellow, terminal portion of femora, tibiae and tarsi sepia to black. Pronotum, abdomen and elytra saffron yellow to terra-cotta, apex of each elytron black with dark blue metallic tinge.

**Figures 2–9.**
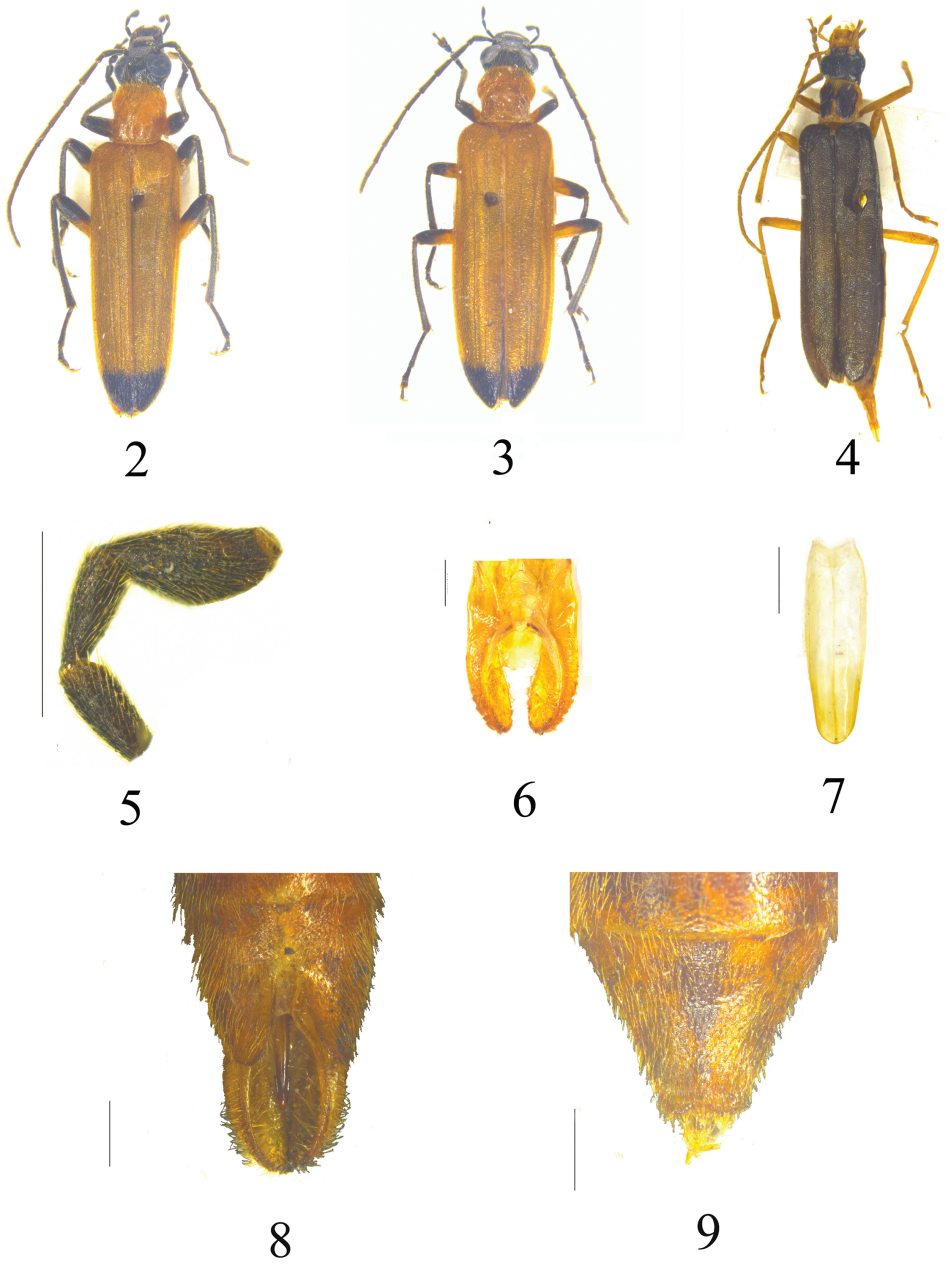
*Nacerdes (Xanthochroa) arcuata* sp. n. (2–3, 5–9) and *Nacerdes (Xanthochroa) potanini* (4). **2** Habitus, male **3** Habitus, female **4** Habitus, female **5** Maxillary palp **6** Projections of urite VIII **7** Tegmen in dorsal view **8** Last sternite, male **9** Last sternite, female. Scale bars = 0.5 mm.

**Figures 10–13.**
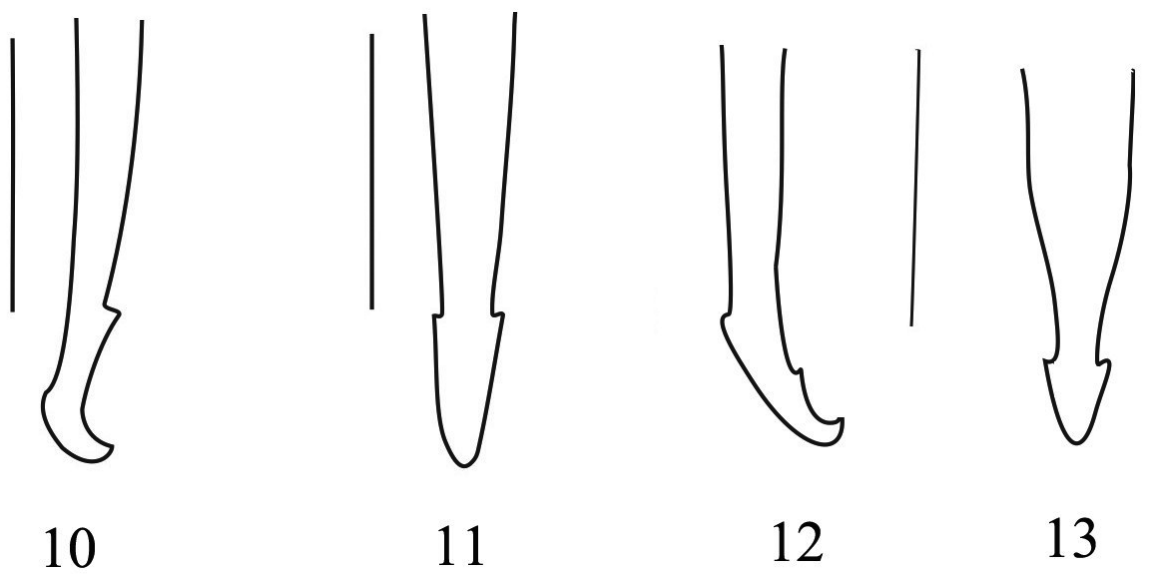
Aedeagal apicale. **10** *Nacerdes (Xanthochroa) arcuata* sp. n. in lateral view **11** *Nacerdes (Xanthochroa) arcuata* sp. n. in dorsal view **12** *Nacerdes (Xanthochroa) hiromichii* in lateral view **13** *Nacerdes (Xanthochroa) hiromichii* in dorsal view; (Figures 12–13 from Švihla 1998). Scale bars = 0.5 mm.

**Male** (Fig. 2). Eyes large, protruding, head across eyes slightly wider than pronotum, frons between eyes 1.8 times as wide as length of antennomere II. Maxillary palp as in Fig. 5. Antennae slightly exceed 1/2 of elytral length, antennomere I more than twice as long as antennomere II, as long as antennomere III, dorsal surface of head and pronotum finely and sparsely punctate and yellow pubescence. Pronotum moderately longer than wide, slightly cordiform, both anterior and posterior pronotal depressions slightly to moderately developed, anterior margin straight, anterior corners rounded, lateral margins moderately sinuate, posterior corners obtuse, posterior margin moderately straight. Elytra nearly parallel-sided, matt, only their dark blue apex lustrous, elytra about four times longer than wide, elytral nervation developed. Both pygidium and last sternite subtriangular, pygidium rounded apically, last sternite incised to 2/3 of its length as in Fig. 8, projections of urite VIII as in Fig. 6. Tegmen (Fig. 7) slightly longer than half of aedeagus, aedeagal apicale as in Figs 10–11.

**Female** (Fig. 3). Head across eyes as wide as pronotum, frons between eyes twice as wide as length of antennomere II, antennae almost reach elytral midlength, pronotum as long as wide, last abdominal segments as in Fig. 9.

##### Distribution.

China: Anhui.

#### Nacerdes (Xanthochroa) hiromichii

Taxon classificationAnimaliaColeopteraOedemeridae

Švihla, 2004

Figs 12–13

Xanthochroa apicalis Kôno, 1932: 141.
Nacerdes (Xanthochroa) apicalis Švihla, 1998: 53.
Nacerdes (Xanthochroa) hiromichii Švihla, 2004: 72; Švihla 2008: 364.

##### Distribution.

China: Taiwan.

#### Nacerdes (Xanthochroa) potanini

Taxon classificationAnimaliaColeopteraOedemeridae

Ganglbauer, 1890

Fig. 4

Xanthochroa potanini Ganglbauer, 1890: 36
Nacerdes (Xanthochroa) potanini Švihla, 1998: 54; Švihla 2008: 364.

##### Material examined.

1♀ (MHBU): China, Gansu, Wen County, Huangtuling, alt. 1,505 m, 33°00'14.85"N, 105°00'41.55"E, 8.vii.2003, Yi-Bin Ba & Yang Yu leg.; 4 ♀♀ (MHBU): China, Sichuan, Jiuzhaigou, alt. 2,457 m, 33°15'35.46"N, 103°55'06.40"E, 7–15.viii.2002, Ming Bai & Jian-Feng Wang leg.

##### Distribution.

China: Sichuan, Gansu.

## Supplementary Material

XML Treatment for Nacerdes (Xanthochroa) arcuata

XML Treatment for Nacerdes (Xanthochroa) hiromichii

XML Treatment for Nacerdes (Xanthochroa) potanini
